# Comparative evaluation of some flavonoids and tocopherol acetate against the systemic toxicity induced by sulphur mustard

**DOI:** 10.4103/0253-7613.42304

**Published:** 2008-06

**Authors:** R. Vijayaraghavan, Anshoo Gautam, Manoj Sharma, H.T. Satish, S.C. Pant, K. Ganesan

**Affiliations:** Defense Research and Development Establishment, Jhansi Road, Gwalior - 474 002, India

**Keywords:** Gossypin, *Hippophae rhamnoides* flavone, oxidative stress, quercetin, sulphur mustard

## Abstract

**Objective:**

To evaluate the protective value of quercetin, gossypin, *Hippophae rhamnoides* (HR) flavone and tocopherol acetate against the systemic toxicity of percutaneously administered sulphur mustard (SM) in mice.

**Materials and Methods:**

Quercetin, gossypin, HR flavone or tocopherol acetate (200 mg/kg, i.p.) were administered just before percutaneous administration of SM and protection against the SM lethality was evaluated. In another experiment quercetin, gossypin, HR flavone or tocopherol acetate were administered against 2 LD_50_ SM. The animals were sacrificed seven days post SM administration and various biochemical parameters were estimated.

**Results:**

The protection against the lethality of SM was very good with the flavonoids (quercetin = 4.7 folds; gossypin = 6.7 folds and HR flavone = 5.6 folds), compared to no protection with tocopherol acetate (0.7 fold). SM (2 LD_50_) showed decrease in reduced and oxidised glutathione (GSH and GSSG) levels, and an increase in malondialdehyde level (MDA). Oxidative stress enzymes like glutathione peroxidase, glutathione reductase and superoxide dismutase were significantly decreased. The total antioxidant status was also significantly decreased. Additionally, there was a significant increase in red blood corpuscles and hemoglobin content. All the flavonoids significantly protected the GSH, GSSG and MDA, and also the hematological variables. Tocopherol acetate failed to offer any protection in those parameters. Gossypin protected glutathione peroxidase, while HR flavone protected both glutathione reductase and glutathione peroxidase significantly. The decrease in body weight induced by SM and the histological lesions in liver and spleen were also significantly protected by the flavonoids but not by tocopherol acetate.

**Conclusion:**

The present study supports that SM induces oxidative stress and flavonoids are promising cytoprotectants against this toxic effect.

## Introduction

Sulphur mustard (SM) chemically, bis 2-chloroethyl sulphide is listed in the Schedule 1 of the Chemical Weapons Convention (CWC). One of the important molecular targets of SM is DNA. SM damages the DNA mainly by alkylating and cross linking the purine bases.[1] At the cellular level, SM causes cytostatis, mutation and slow cell death. Eyes, skin and the respiratory tract are the main target organs of SM.[2] In the recent past, substantial efforts have been made in developing pharmacological strategies against the toxic effects of SM. All these studies were aimed at preventing or reversing SM alkylated critical cell targets, improve calcium regulation, protect cell mediated biochemical disruptions or prevent cytotoxicity.[3,4] Though lots of compounds have shown good prophylactic as well as therapeutic protection *in vitro*, their *in vivo* efficacy has not been proved.[5–7] Till today there is no conclusive therapy to counteract the systemic toxicity of SM. Better understanding of the cellular mechanism by which SM causes cytotoxicity will aid the search for specific cytoprotectants. After the CWC came into force, destruction of SM is being carried out by the declared state parties. An effective prophylactic or therapeutic antidote in addition to the personal protection is required for this purpose.[8] Taking into consideration the increasing terrorist activities and also that the chemical weapons can be used clandestinely, drug development against SM is required not only for defence personnel but also for the civilian usage.

One approach to mitigate SM toxicity is cellular glutathione replenishment. Glutathione, cysteine and other endogenous thiols are able to reduce toxic effects of SM and other xenobiotics by conjugation reaction. Cysteine and glutathione have been tried against SM toxicity *in vitro* showing good protection.[9] N-acetyl cysteine has also been shown to increase cellular cysteine levels available for GSH synthesis.[10] However an *in vivo* protection of these compounds has not yet been reported. Other potential treatment strategies include barrier creams,[11] reagents to improve maintenance of cellular NAD^+^ levels,[12–14] antihistamines,[15] non-steroidal anti-inflammatory agents,[4,16] SM scavengers,[16] Poly ADP ribose polymerase (PARP) inhibitors[14,17] and arginine analogues.[7] Some PARP inhibitors and povidone iodine ointment were found to be effective as post treatment also.[18] Out of all these compounds, only povidone iodine ointment and some anti-inflammatory agents have been tested *in vivo*. So far, however, there is no drug that can protect the animals from the systemic toxicity of SM. Effective chemical decontaminants are reported viz., S-330 and CC2.[8,19] Amifostine (WR2721) originally developed by the United States army as a radioprotector, has shown appreciable protection against the toxicity of antineoplastic alkylating agents. DRDE-07, an aminoalkyl aminoaryl sulphide developed from structural modifications of amifostine showed better protection then amifostine *in vitro* and *in vivo*.[20–22]

Flavonoids are polyphenolic phytochemicals that are ubiquitously present in plants and are well studied in a variety of conditions. Many recent reports are available that show quercetin and gossypin having antioxidant and anticarcinogenic activity. Quercetin is helpful in the recovery of N-diethyl nitrosamine induced carcinogenesis,[23] human leukemia cell,[24] streptozotacin induced diabetes,[25] chronic renal failure and reactive oxygen species (ROS) induced DNA damage.[26] Gossypin is another well-known antioxidant[27,28] reported as an antinociceptive agent.[29,30] Gossypin has protective effect on CCl_4_ induced toxicity in rat hepatocytes.[31] Reports are also available about its anti-inflammatory, antiallergic, and inhibitory action on arachidonic acid metabolism.[32,33] It has been reported that tocopherol acetate (vitamin E) has hepatoprotective and antioxidant properties.[34–36] It also protects against gamma radiation induced gastric and duodenal mucosal injury.[36,37] *Hippophae rhamnoides* (Linn) is a high altitude plant and its berries are highly nutritive. A number of flavones (HR-flavone) are identified in the fruits having beneficial effects in a number of disease states.[38–40] Quercetin and isorhamnetin are the flavonoids present in *H. rhamnoides*.

Since the flavonoids are well tolerated, widely studied and least toxic, we initiated this study on the comparative evaluation with tocopherol acetate against percutaneously administered SM. We have reported earlier that percutaneous administration of SM is more toxic than subcutaneous route and studies on the antidotal efficacy of SM should preferably use the *in vivo* methods.[41,42]

## Materials and Methods

Chemicals: SM was synthesized in the Synthetic Chemistry Department and was found to be 99% pure by gas chromatographic analysis. O-pthalaldehyde (OPT) oxidized and reduced glutathione, 4′6-diamidino-2-phenylindole (DAPI) were purchased from Sigma Chemical Company (USA). Other chemicals of high purity were from Qualigen (India) or Merck (India). Glutathione peroxidase (GP), glutathione reductase (GR), superoxide dismutase (SOD), and total antioxidant kit were purchased from Cal Biochem (India).

Quercetin and gossypin were purchased from Aldrich (USA) and Bioorganics, (India) respectively. *Hippophae rhamnoides* flavone (HR-flavone) is a mixture of flavonoids isolated from the fruit (Beiging Jianghe Sea Buckthorn Company, Beiging, China). Tocopherol acetate (vitamin E) was procured from Fluka (USA).

Animals: Randomly bred Swiss female mice weighing between 25-30 g from Defence Research and Development Establishment's (DRDE) animal facility were used for the study. The animals were housed in polypropylene cages under controlled environmental conditions with free access to food (Ashirwad Ltd, India) and water. The care and maintenance of animals were as per the approved guidelines of the Committee for the Purpose of Control and Supervision of Experiments on Animals (CPCSEA, India). A day before percutaneous exposure of SM, hair on the back of the animals was closely clipped using a pair of scissors. Food and water were withheld two hours prior to the experiment. The Institutional Animal Ethical Committee approved this study.

Protection of flavonoids against lethal doses of SM: The flavonoids were dissolved in PEG-300 and tocopherol acetate was dissolved in olive oil. SM was diluted in PEG-300 and applied dermally (percutaneous route). Logarithmic doses of SM were used and the flavonoids and tocopherol acetate (200 mg/kg) were administered i.p., just before the SM administration (simultaneously). Four animals per group were used. The animals were weighed daily and were observed for 14 days and LD_50_ determinations were carried out as per moving average method.[43] For each LD_50_ determination 3 to 4 groups were used. Protective index (PI) was determined as a ratio of LD_50_ of SM after treatment to LD_50_ of SM without treatment.

Protective efficacy of flavonoids against SM induced biochemical and histological changes: A single dose of SM (2 LD_50_; 19.4 mg/kg) was administered percutaneously. The flavonoids and the tocopherol acetate were administered at a dose of 200 mg/kg, i.p., just before SM administration. A total of six groups were used and each group consisted of four animals. The control group was administered with PEG-300, i.p. and applied dermally with PEG-300. The animals were weighed daily. Seven days after SM administration, the animals were anesthetized with ether and blood was collected from orbital plexus in heparinized tubes. The animals were then sacrificed by cervical dislocation and liver and spleen were dissected out for biochemical and histological evaluations.

Biochemical and histological evaluations: The liver and spleen were dissected out, freed from adhering tissues and weighed. The organ to body weight indices were calculated following the formula OBWI = 100 × (organ weight / body weight). Pieces of liver tissue were taken for GSH, GSSG and MDA estimation. Fluorimetric method of Hisin and Hilf[44] was used for the estimation of GSH and GSSG. Briefly, 150 mg of liver tissue was homogenized in phosphate EDTA buffer. The homogenate was centrifuged at 10,000 g at 4°C for 15 min. To 0.25 ml of supernatant, 100 *μ*l of 1 mg/ml fluorescent dye OPT was added and after 15 min of incubation at room temperature, readings were taken at 420 nm emission and 350 nm excitation. Hepatic lipid peroxidation was determined by measuring the level of MDA according to the method of Buege and Aust.[45] 100 mg of liver was directly homogenized in 5 ml of thiobarbituric acid reagent and boiled for 30 min. The contents of the tubes were cooled, centrifuged and absorbance of the clear supernatant was measured at 535 nm. The amount of MDA formed was calculated using a molar extinction coefficient of 1.58 × 10^5^/M per cm.

The blood was used for the estimation of RBC and Hb, using Backman Coulter Cell Counter (USA). A portion of the blood was centrifuged to separate plasma. Total antioxidant status of plasma was estimated using Cal Biochem Kit. 100 mg of liver samples each were used for the estimation of GP, GR and SOD, using Cal Biochem Kit.

Liver and spleen samples were fixed in 10% neutral buffered formalin solution. After proper fixation, small pieces were processed by dehydration and embedded in paraffin wax. Multiple sections of 5 - 6 *μ*m thickness were prepared and stained with hematoxylin and eosin for light microscopic observation.[46] Lesions were marked and compared with that of control. The severity of lesions was characterized using LEICA - Qwin - 500 Image Analyser (Leica Orthoplan, Germany).

### Statistical analysis

All the variables were analyzed by one-way ANOVA followed by Bonferroni multiple comparisons test. A probability of < 0.05 is taken as statistically significant. SigmaStat (SPSS Inc., USA) was used for statistical calculations.

## Results

The animals administered with SM died at varying intervals depending upon the dose. The estimated LD_50_ of SM was 9.7 mg/kg. The survival of mice treated with quercetin, gossypin and HR flavone was better than that of tocopherol acetate. The PI of quercetin, gossypin and HR flavone was 4.7, 6.7 and 5.6 folds respectively, compared to 0.7 fold of tocopherol acetate [Table 1]. The body weight decreased progressively when 2 LD_50_ of SM was administered percutaneously. This body weight decrease (7 days post SM administration) was significantly protected by quercetin, gossypin and HR-Flavone but not by tocopherol acetate [Table 2]. Effects of flavonoids and tocopherol acetate on OBWI of liver and spleen are also shown in Table 2. Significant decrease in liver and spleen weights was observed in SM administered animals. SM induced decrease in OBWI of spleen was protected by flavonoids but not by tocopherol acetate.

**Table 1:** Protective efficacy of different flavonoids as compared to tocopherol acetate against percutaneously administered sulphur mustard (SM)

*Groups*	*LD50 (mg/kg)*	*Confidence limit (mg/kg)*	*PI*
*SM only*	*9.7*	*6.1 - 15.2*	*-*
*+ Quercetin*	*45.9*	*25.3 - 83.7*	*4.7*
*+ Gossypin*	*65.0*	*35.7 - 118.32*	*6.7*
*+ HR- Flavone*	*54.7*	*28.8 - 103.6*	*5.6*
*+ Tocopherol Acetate*	*6.7*	*4.8 - 9.7*	*0.7*

Flavonoids and tocopherol acetate (200 mg/kg; i.p.) were administered simultaneously with SM. Protective Index (PI) = Ratio of LD50 with treatment to LD50 without treatment

**Table 2:** Effect of flavonoids and tocopherol acetate on body weight, and liver and spleen weights

*Groups*	*Body weight (%)*	*Liver weight (%)*	*Spleen weight (%)*
Control	101.5 ± 2.0^b^	6.22 ± 0.86^b^	0.527 ± 0.03
SM only	68.6 ± 5.9^a^	4.43± 0.07^a^	0.328 ± 0.03^a^
+ Quercetin	91.8 ± 5.6^b^	5.04 ± 0.02	0.662 ± 0.03^b^
+ Gossypin	98.3 ± 1.4^b^	4.19 ± 0.15^a^	0.665 ± 0.03^b^
+ HR- Flavone	90.5 ± 7.3^b^	5.37 ± 0.32	0.785 ± 0.10^a^^b^
+ Tocopherol Acetate	60.3 ± 3.5^a^	3.97 ± 0.31^a^	0.307 ± 0.01^a^

Mean ± SE (n = 4) have been shown.

a Significantly different from control group

b Significantly different from SM group. Flavonoids and tocopherol acetate (200 mg/kg, i.p.) were administered simultaneously with SM. The animals were sacrificed 7 days post SM administration. Liver and spleen weight are expressed per 100 g body weight of the animal.

**Figure 1:**
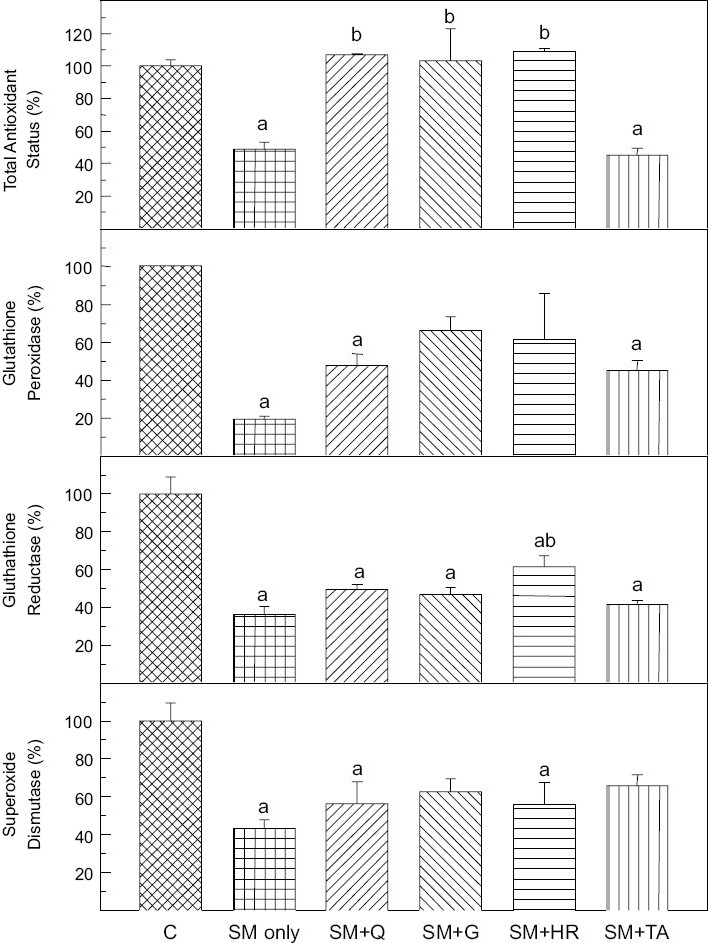
Effect of flavonoids in comparison to tocopherol acetate (200 mg/kg, i.p.) against percutaneously administered SM (2 LD50). Q = quercetin; G = gossypin; HR = H. rhamnoides flavone; TA = tocopherol acetate. Mean ± SE (n = 4); Flavonoids and tocopherol acetate were administered simultaneously with SM. The animals were sacrificed seven days post SM administration. a - Significantly different from control group; b - Significantly different from SM group. Control values:

The effect of various flavonoids and tocopherol acetate on oxidative stress markers is given in Table 3. Significant reduction in hepatic GSH and GSSG content was observed when compared to control. In SM treated group, 38.4% and 43.2% reduction in GSH and GSSG was observed, respectively. This reduction was protected by all flavonoids but not by tocopherol acetate. There was a significant increase in MDA level in SM group compared to control and this was protected by flavonoids as well as by tocopherol acetate. Significant increase in RBC count and hemoglobin content was also observed after percutaneous administration of SM. This elevation in RBC count and hemoglobin content was protected by flavonoids but not by tocopherol acetate [Table 3]. Total antioxidant status was significantly reduced (49.0 % of control) after SM administration. It was significantly protected by quercetin, gossypin and HR flavone but not by tocopherol acetate. Various oxidative stress marker enzymes were also affected by SM administration. GP, GR and SOD activities were significantly reduced after SM administration. There was a marginal improvement in the level of the oxidative stress enzymes with the flavonoids that was insignificant statistically [Figure 1].

**Table 3:** Changes in biochemical and hematological variables after treatment with different fl avonoids and tocopherol acetate against percutaneously administered Sulphur mustard (SM)

*Group^∗^*	*GSH (%)*	*GSSG (%)*	*MDA (%)*	*RBC (%)*	*Hb (%)*
Control	100.0^b^	100.1^b^	100.0^b^	110.0^b^	100.0^b^
	± 3.1	± 2.4	± 6.9	± 4.4	± 2.2
SM only	38.4^a^	43.2^a^	152.6^a^	157.9^a^	156.3^a^
	± 6.1	± 8.2	± 22.2	± 15.2	± 6.6
+ Quercetin	76.4^b^	72.5^a^^b^	108.0^b^	118.5^b^	92.5^b^
	± 7.9	± 8.6	± 5.4	± 7.0	± 13.2
+ Gossypin	80.5^b^	77.7^b^	103.8^b^	106.4^b^	96.7^b^
	± 10.5	± 8.8	± 4.2	± 2.7	± 2.8
+ HR- Flavone	76.8^b^	81.9^b^	104.3^b^	111.2^b^	100.2^b^
	± 9.3	± 9.4	± 2.8	± 6.7	± 6.3
+ Tocopherol acetate	46.5^a^	48.8^a^	115.2^b^	153.5^a^	140.7^a^
	± 0.2	± 0.4	± 7.6	± 2.4	± 0.5

Mean ± SE (n = 4) have been shown.

a Significantly different from control group

b Significantly different from SM group. Flavonoids and tocopherol acetate (200 mg/kg, i.p.) were administered simultaneously with SM. The animals were sacrificed 7 days post SM administration. Control values GSH = 7.2 ± 0.4 µmoles/gm of tissue, GSSG = 8.6 ± 0.4 µmoles/gm of tissue, MDa = 4.43 ± 0.5 nmoles/gm of tissue, RBC = 8.5 ± 0.1 X10^6^ cells/mm^3^, Hb = 13.0 ± 0.5 g/dl

**Table:** 

Antioxidant status (plasma)	=	139.0 ± 4.9 μM
Glutathione Peroxidase (liver)	=	747 ± 19 nanomoles of NADPH/min/mg protein
Glutathione Reductase (liver)	=	43.5 ± 1.2 nanomoles NADPH/min/mg protein
Superoxide Dismutase (liver)	=	5.60 ± 0.56 U/mg protein

**Figure 2:**
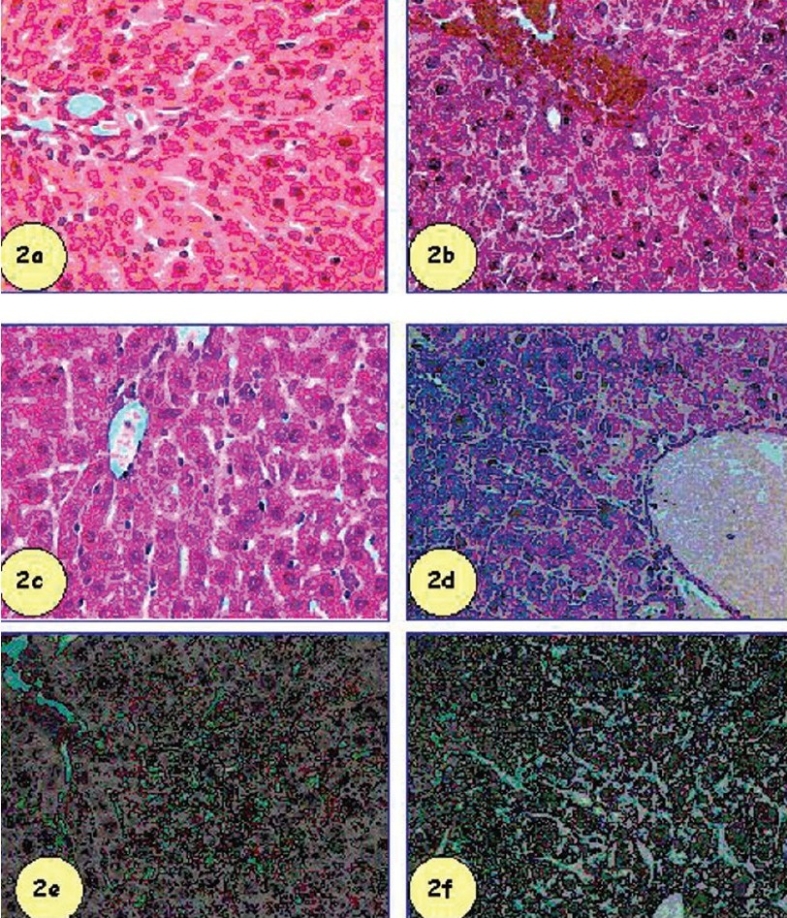
Effect of flavonoids in comparison to tocopherol acetate (200 mg/kg, i.p.) against percutaneously administered SM (2 LD50) on mice liver. Flavonoids and tocopherol acetate were administered simultaneously with SM. The animals were sacrificed seven days post SM administration. Q = quercetin; G = gossypin; HR = H. rhamnoides flavone; TA = tocopherol acetate. H&E, ×100.
Control liver showing normal hepatic parenchyma, hepatic lobules and hepatocytes,SM administered mice liver (2 LD50) showing granulovacuolar degeneration and perinuclear clumping of cytoplasm,Quercetin administration with SM, showing reduced hepatic lesions,Gossypin administration with SM showing lesser magnitude of hepatic lesions,HR-flavone administration with SM showing protection of hepatic degeneration,Tocopherol acetate administration with SM showing marginal protection.

SM administration by percutaneous route (2 LD_50_) showed significant lesions in hepatic tissues that were granulovascular degeneration of hepatocytes, perinuclear clumping of cytoplasm and hyperactivation of kuffer cells. Liver cells of mice after SM administration, showed moderate centri lobular necrosis/apoptosis with occasional diffuse vacuolar degeneration of hepatocytes in midzonal area along with severe congestion and hemorrhage. No such degeneration was observed in quercetin, gossypin and HR-flavone pre-treatment groups. Severe hepatic lipidosis and accumulation of fibrinoid debries were observed in tocopherol treated mice with SM intoxication. Inflammatory cell infilteration was also observed in this group [Figure 2].

**Figure 3:**
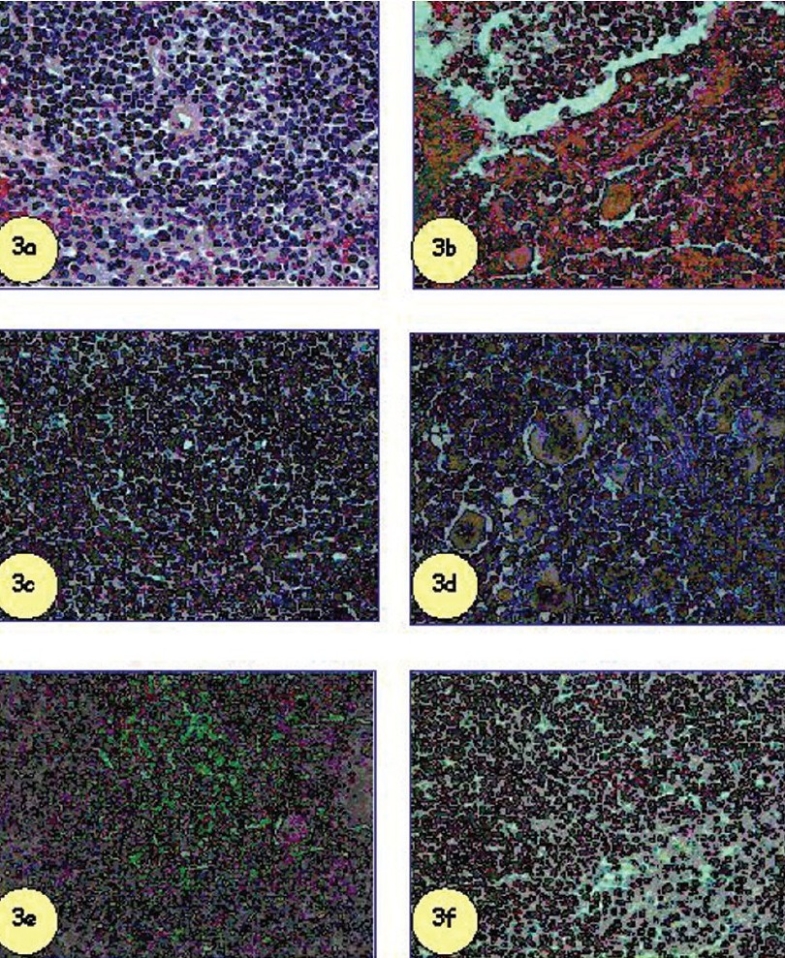
Effect of flavonoids in comparison to tocopherol acetate (200 mg/kg, i.p.) against percutaneously administered SM (2 LD50) on mice spleen. Flavonoids and tocopherol acetate were administered simultaneously with SM. The animals were sacrificed seven days post SM administration. Q = quercetin; G = gossypin; HR = H. rhamnoides flavone; TA = tocopherol acetate. H&E, ×100.
Control spleen showing normal splenic parenchyma germinal centre, red and white pulp,SM administered mice spleen (2 LD50) showing congestion, hypocellularity and parenchymal degeneration,Quercetin administration with SM showing protection with lesser hypocellularity,Gossypin administration with SM showing lesser magnitude of splenic degeneration,HR-flavone administration with SM showing minimal lesions,Tocopherol acetate administration with SM showing marginal protection.

Histopathological examination showed degeneration and necrosis when SM was administered percutaneously (2 LD_50_). No alteration was observed when quercetin, gossypin, or HR-flavone pre-treatment was given. Megakaryocytes and degeneration of periarteriolar lymphatic sheath was seen when tocopherol acetate was administered simultaneously with SM [Figure 3].

## Discussion

A number of mechanisms have been proposed for the toxic effects of SM and out of that the oxidative stress mediated mechanism is gaining importance.[47–50] In the aqueous medium, SM is capable of undergoing nucleophilic substitution reactions to initiate free radicals either directly or indirectly and also generate reactive oxygen intermediates.[48] GSH, a cysteine containing tripeptide plays an important role in the detoxification of xenobiotics and in the scavenging of reactive species and free radicals. It accounts for almost 90% of cellular nonprotein thiols.[51] GSH levels are reduced in human peripheral lymphocytes when incubated with SM.[52] Davison *et al*,[53] reported that the major urinary metabolites of SM are glutathione conjugates. Decrease in the level of GSH has been reported *in vivo* by several investigators as a toxic effect of SM and the monofunctional analogue chloroethylethyl sulphide.[47,48,54]

Significant reduction of GSH due to SM is indicative of oxidative stress and cellular damage. Direct interaction of GSH with SM is also possible. Being electrophile in nature, SM has high affinity towards sulphhydryls groups and thus it depletes GSH in the body. The decrease was significantly protected by flavonoids but not by tocopherol acetate. This may be due to the nucleophilic interaction of the electrophile. In this study the flavonoids were administered simultaneously with SM. It has already been reported that flavonoids protect only when they are administered as a pre-treatment or simultaneously that means that flavonoids should be available before the SM metabolite reach the target molecule. In such a case flavonoids donate its hydroxyl group to free radicals sparing GSH. The increase in the level of MDA after SM administration is recovered by all the flavonoids and tocopherol acetate. Usually lipid peroxidation takes place in the presence of iron molecule by the reactive oxygen species. Flavonoids are known to chelate iron, thereby removing the causal factor for the development of free radical. Quercetin in particular is known for its iron chelating and iron stablizing property. Direct inhibition of lipid peroxidation is another protection measure of flavonoids. This may be the reason for the dose of the flavonoids to be fairly high. Significant recovery in MDA levels is due to the antioxidant activity of tocopherol acetate. Other reports are also available that tocopherol acetate helps in the recovery of lipid peroxidation.[55,56] Due to generation of reactive oxygen species by SM, the membrane loses its integrity and fluidity. The endothelial cells fail to retain plasma in blood, and the viscosity and density of blood increases, resulting in increase in RBC count and hemoglobin content. Many reports are available that RBC count and hemoglobin content are increased after SM exposure.[57,58] To compensate, the blood from spleen also enters the circulation resulting in the shrinkage of the spleen. All these effects are significantly protected by flavonoids and not by tocopherol.

SOD (Superoxide dismutase) is a family of metalloenzymes that convert O2^.-^ to H_2_O_2_. Under normal circumstances formation of superoxide anion is under the control by SOD enzymes. It was also reported that superoxide anion is intimately involved with the inflammatory response.[59] A significant reduction was found in its activity after seven days post SM administration and it was not recovered by any antioxidant. GR is also decreased significantly and was not recovered by the flavonoids and tocopherol acetate. GP is a selenium containing enzyme that utilises GSH as a cofactor and catalyses the oxidation of GSH to GSSG at the expense of H_2_O_2_. SM decreased the activity of GP, which was protected by gossypin and HR-flavone, but not by quercetin and tocopherol acetate. GP, GR and SOD are generally known to be involved in either regenerating GSSG or regulating cellular redox state. A decrease in GSH and GSSG has been observed that is not usually found in oxidative stress states. This means that SM active metabolites may be responsible to interact directly with these molecules and alter its activity. Post-translational modifications are also possible to the change of activity of GSH, GSSG, GP, GR, and SOD rather than affecting its cascade. SM also causes multi-organ failure and its effect need not necessarily be on gene level. These findings show that ROS is not the only mechanism to explain SM toxicity but some other factors may also be responsible. Flavonoids may directly interact with active molecule of SM to offer protection. This also explains that flavonoids are effective in pre-treatment as well as simultaneous treatment but not as posttreatment.[60]

Our previous findings suggested that dermally applied SM affected liver and spleen. In the present study also, there was a significant reduction in OBWI of liver and spleen in mice administered with SM at a dose of 2 LD_50_. This may be due to the effect of SM on fast growing cells i.e., liver and spleen. Quercetin and HR-flvone marginally protected the reduction in OBWI of liver. Liver is the main organ for detoxification and the beneficial effects of flavonoids may be related to their hepatoprotective potential. The reduction in the spleen weight was significantly protected by all the three flavonoids but only marginally by tocopherol acetate. The histopathologic changes in liver and spleen of percutaneously applied SM were due to the systemic toxicity. It was recovered by quercetin, gossypin and HR- flavone. Flavonoids may protect the toxicity of SM due to their anti-inflammatory property or antioxidant property. No protection was observed in tocopherol acetate treated groups. This strongly suggests the hepatoprotective effect of the flavonoids. Reports are available that Vitamin E is more effective than Vitamin C in restoration of alteration caused by CCl_4_.[34,56] Significant protection has been observed in halothane induced oxidative stress and CdCl_2_ induced toxicity.[55] Vitamin E per se is not sufficient to give antioxidant property, but it is effective as Vitamin E - selenium combination.[36] Tocopherol acetate may be effective as an antioxidant with the supplement of Vitamin C or selenium.[35,37]

The mortality depends upon the dose applied and the LD_50_ varies with the observation period. Our earlier reported LD_50_ values varied from 5.6 to 8.1 mg/kg, but in the present study it is 9.7 mg/kg. A number of factors may be responsible for these variations viz, skin texture of animals and the environmental conditions. Significant protection was observed when flavonoids were administered intraperitoneally, in PEG-300 simultaneously with SM exposure or as pre-treatment.[60] Flavonoids offered good protection against SM toxicity but the protection was very less in the case of tocopherol acetate. The reason might be the hydroxyl group located in 3′ position of all these flavonoids are highly reactive to interact with electrophilic molecule and no such group is available in tocopherol acetate. Tocopherol acetate acts as an antioxidant and breaks free radical chain reactions as a result of its ability to transfer a phenolic hydrogen to a peroxyl free radical of a peroxidised poly unsaturated fatty acids. The body weight of animals decreased progressively and in some cases it was 60.0% of the initial body weight. This was partially due to the reduced food and water intake as a result of the toxic effects of SM. The protection against SM induced weight loss was observed when flavonoids were administered but no such protection was observed when tocopherol acetate was administered. This is due to the interaction of flavonoids with SM preventing the early responses in the body.

Inflammatory response plays a vital role in SM toxicity.[3,61] Arachidonic acid pathway is one of the key biomolecule pathways involved in inflammation and the release of arachidonic acid is a starting point for inflammatory response. Flavonoids inhibit arachidonic acid metabolism resulting in its anti-inflammatory action. Neutrophils containing lipoxygenase create chemotactic compounds from arachidonic acid. This also provokes the release of cytokines. Anti-inflammatory action of tocopherol acetate is not reported so far and this may be another reason for its lack of protection in SM toxicity. SM is a potent cytotoxic chemical and this study supports the strong cytoprotective effect of flavonoids.
